# Extensive fibrotic wrapping of the heart: a rare echocardiographic diagnosis

**DOI:** 10.1186/s12947-022-00289-y

**Published:** 2022-07-25

**Authors:** Wei Jiang, Lili Xu, Xiaojuan Guo, Yidan Li, Xiuzhang Lv

**Affiliations:** 1 Department of Echocardiography, Beijing Chao Yang Hospital, Capital Medical University, No. 8 Gongti South Road, Chaoyang District, Beijing, China; 2 Department of Respiratory and Critical Care Medicine, Beijing Chao Yang Hospital, Capital Medical University, Beijing, China; 3 Department of Radiology, Beijing Chao Yang Hospital, Capital Medical University, Beijing, China

**Keywords:** Fibrosing mediastinitis, Echocardiography, Diffuse mediastinal infiltration, Idiopathic

## Abstract

**Background:**

Fibrosing mediastinitis (FM) is considered a benign disease, but it can be fatal if progression leads to compression of the hilum of the lungs or invasion of the heart. Echocardiographic reports of this disease are very rare.

**Case presentation:**

We present a 14-year-old male patient whose non-enhanced chest computed tomography showed unclear soft-tissue dense lesions in the anterior superior mediastinum. Echocardiography showed the heart was extensively wrapped by soft tissue lesions. The histology confirmed FM.

**Conclusions:**

When FM affects the heart, echocardiography can help to characterize the disease and aid in the diagnosis. Echocardiography should be considered an important tool to follow the progression of this disease and guide the therapeutic approach.

**Supplementary Information:**

The online version contains supplementary material available at 10.1186/s12947-022-00289-y.

## Background

Fibrosing mediastinitis (FM) is a rare and benign disease, but progression can potentially be fatal. It is characterized by compression and obstruction from hyperplastic fibrous tissue that encloses the hilar structure of the lungs, and often needs to be differentiated from malignancy [1]. Histopathology is the gold standard for diagnosis, but imaging findings can aid in clinical diagnosis. The role of echocardiography in diagnosis is uncertain, because there are few reports of its application in the setting of this disease. Echocardiographic results were reported in individual case reports of FM [2, 3], but there were no previous presentations or detailed descriptions of imaging results, and there has been no focus on the use of echocardiography as a diagnostic tool for patients with FM.

We report a case of idiopathic FM with extensive cardiac involvement. We examined the patient using echocardiography and performed follow-up over 4 years. To our knowledge, this is the first description of the application of echocardiography in FM.

## Case presentation

**Fig. 1:**
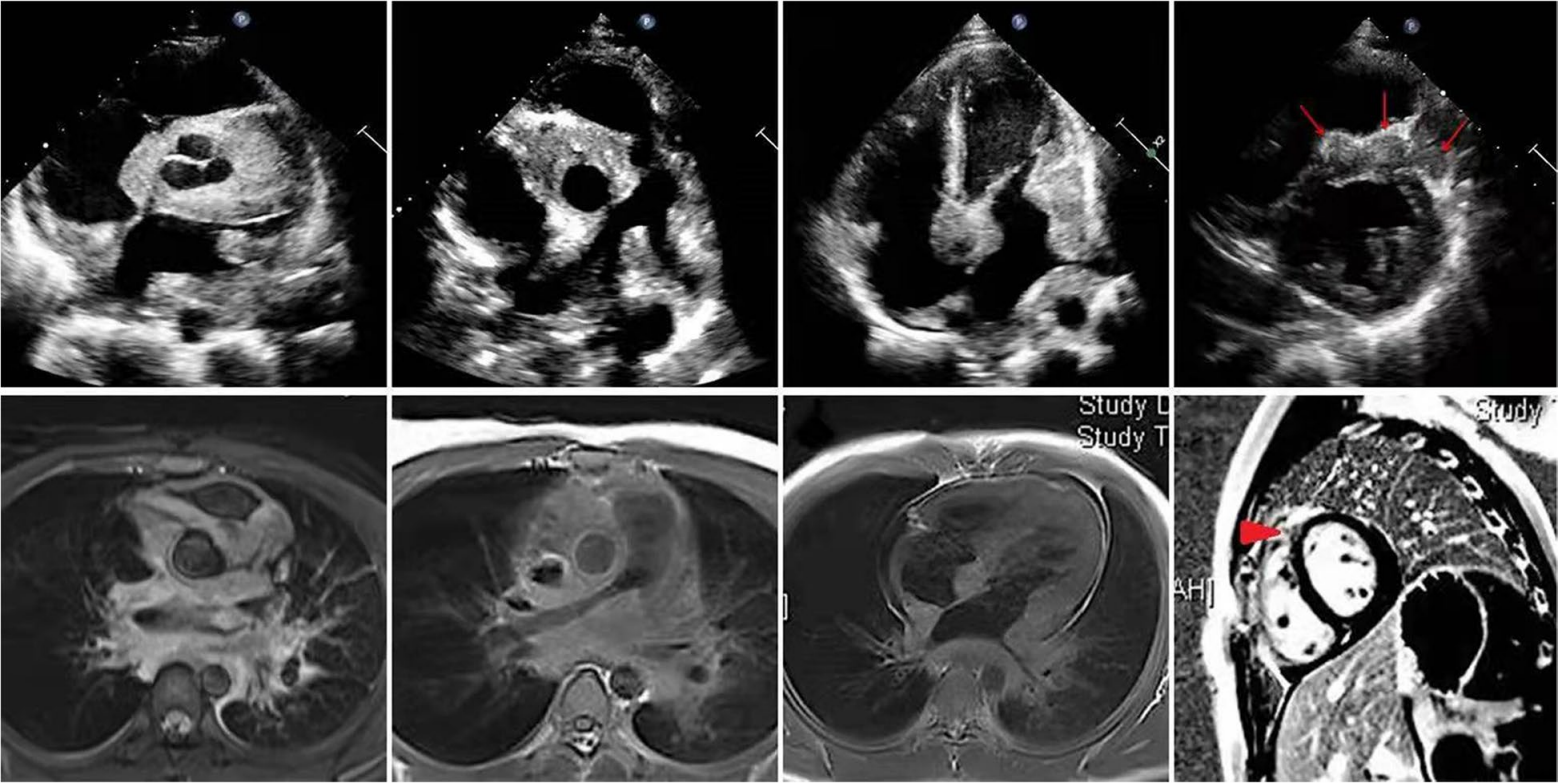
Echocardiography and enhanced cardiac MRI at admission showed diffuse lesions invading the heart and infiltration of the myocardium (arrow)

A 14-year-old male patient presented with chest pain for 2 months and hemoptysis for 3 days. There was no clinically relevant medical history, and no family history of malignancies or autoimmune diseases. The patient showed no signs of infection and the physical examination results were normal. A non-enhanced chest computed tomography (CT) showed a soft tissue mass in the anterior superior mediastinum, but the structure was not clear. Laboratory tests showed the C-reactive protein (CRP) was slightly elevated (0.94 mg/dL, normal: < 0.8 mg/dL) and the erythrocyte sedimentation rate (ESR) was within the normal range (9 mm/h, normal: 2–15 mm/h). There were no meaningful findings in the laboratory indicators of infectious diseases, tumors, or autoimmune diseases (Table 1). Echocardiography showed that the left and right pulmonary arteries and pulmonary veins, aortic root, proximal left and right coronary arteries, left and right atria, base of the left ventricle, and both sides of the atrial septum were surrounded by masses with medium level of echogenicity, some of them not clearly separated from the myocardium. The pulmonary arteries and pulmonary veins were narrowed, and blood flow velocity was increased in the left and right pulmonary arteries. There was a mild enlargement of right heart chambers, mild to moderate tricuspid regurgitation with a gradient of 53 mmHg, and small pericardial effusion (Table 2). Cardiac magnetic resonance imaging (MRI) indicated slightly longer T1 and longer T2 signal lesions in the same area as the echocardiographic abnormalities. We considered inflammatory granulomatous lesions and malignant tumors in the differential diagnosis (Fig. 1). Because diagnosis could not be determined based on these findings, we scheduled the patient for thoracoscopy and biopsy of the mediastinum and myocardium.

**Table 1:** Infection markers, tumor markers, and immunological indicators

Infection markers			Tumor markers			Immunological indicators		
Test	Results	Reference range	Test	Results	Reference range	Test	Results	Reference range
PCT, ng/ml	** < 0.05**	< 0.05	SCC, ng/ml	**0.7**	0–1.5	IgG, mg/dl	**1530**	751–1560
G-test, pg/ml	**42.24**	< 100	CEA, ng/ml	**0.46**	< 0.5	IgG4, mg/ml	**122**	80–1400
Sputum acid-fast bacilli	Negative	Negative	CA19-9, U/ml	**10.27**	< 37	Complement C3, mg/ml	**95.7**	79.00–152.00
Germiculture	Negative	Negative	AFP, ng/ml	**2.6**	< 8.1	Complement C4, mg/ml	**21.5**	12.00–36.00
Fungal culture	Negative	Negative	CYFRA21-1, ng/ml	**2.01**	0–2.08	ANA	**1: 100**	< 1: 100
Gene-Xpert	Negative	Negative	CA125, U/ml	**7.2**	< 30.2	Anti-dsDNA	Negative	Negative
			NSE, ng/ml	**14.63**	0–16.3	Anti-Sm	Negative	Negative
			CA724, U/ml	**0.55**	0–8.2	Anti-RNP	Negative	Negative
						Anti-Jo1	Negative	Negative
						Anti-Ro/SSA	Negative	Negative
						Anti-La/SSB	Negative	Negative
						Anti-Scl70	Negative	Negative
						AHA	Negative	Negative
						ANuA	Negative	Negative
						ARPA	Negative	Negative
						ACA	Negative	Negative
						MPO-ANCA, U/ml	**1.8**	< 5
						PR3-ANCA, U/ml	**2.6**	< 5

*PCT* Procalcitonin, *SCC* Squamous cell carcinoma, *CEA* Carcinoembryonic antigen, *CA19-9* Carbohydrate antigen 19–9, *AFP* Alpha-fetoprotein, *CYFRA21-1* Cytokeratin fragment antigen 21–1, *CA125* Cancer antigen 125, *NSE* Neuron-specific enolase, *CA724* Carbohydrate antigen 724, *IgG* Immunoglobulin G, *IgG4* Immunoglobulin G4, *ANA* Antinuclear antibody, *Anti-dsDNA* Anti-double stranded DNA, *AHA* Antihistone antibodies, *ANuA* Antinucleosome antibody, *ARPA* Anti-ribosomal P protein antibodies, *ACA* Anti-centromere antibody, *MPO-ANCA* Myeloperoxidase anti-neutrophil cytoplasmic antibody, *PR3-ANCA* Proteinase 3 anti-neutrophil cytoplasmic antibody

**Table 2:** Echocardiographic parameters at admission and during follow-up

	Admission	3 months	13 months	27 months	50 months
**LV**					
EDD, mm	43	45	44	47	51
EDV, ml	81	92	91	105	126
EF, %	72	72	73	61	65
E, cm/s	97	80	71	51	53
A, cm/s	49	112	61	47	43
**RV**					
RVD, mm	37	35	34	32	38
TAPSE, mm	12.2	NR	28	NR	NR
TIPG, mmHg	53	47	28	23	21
**Great vessels**					
AO, mm	17	20	23	27	28
LPA, mm	8.4	11	13	13	15
RPA, mm	7.5	8.7	11.4	12	12
IVC, mm	20	18	10.5	NR	NR
Blood flow velocity of left pulmonary arteries, cm/s	274	182	117	109	146
Blood flow velocity of right pulmonary arteries, cm/s	320	278	168	158	160
Blood flow velocity of pulmonary veins, cm/s	212	180	129	116	NR
**Lesion area**		reduced	reduced	reduced	basically normal

*LV* Left ventricular, *EDD* End-diastolic diameter, *EDV* End-diastolic volume, *EF* Ejection fraction, *RV* Right ventricular, *RVD* Right ventricular diameter, *TAPSE* Tricuspid annular plane systolic excusion, *TIPG* Tricuspid pressure grade, *AO* Aorta, *LPA* Left pulmonary artery, *RPA* Right pulmonary artery, *IVC* Inferior vena cava, *NR* Not recorded

During thoracoscopy, we observed bulging lesions around the left superior and inferior veins of the left atrium (3 × 2 cm), lateral wall of the left atrium and left ventricular (4 × 3 cm), and the top of the left atrium (3 × 2 cm). The lesions were hard/brittle and bled easily upon touch. The same lesions were present in the transverse pericardial sinus and around the pulmonary arteries. The pathological examination revealed the lesions consisted of hyperplastic fibrous tissues with infiltration of lymphocytes and plasma cells, a blurry lesion boundary, and involvement of the surrounding adipose tissue, with a surrounding of small nerves and blood vessels. There was no evidence of granuloma or malignant cells, and the IgG4 staining test was negative. Fungal culture and acid-fast staining tests were also negative. After multidisciplinary discussion, we made a diagnosis of idiopathic FM based on the highly suggestive radiology, definitive pathological results, and the exclusion of other diagnoses.

**Fig. 2:**
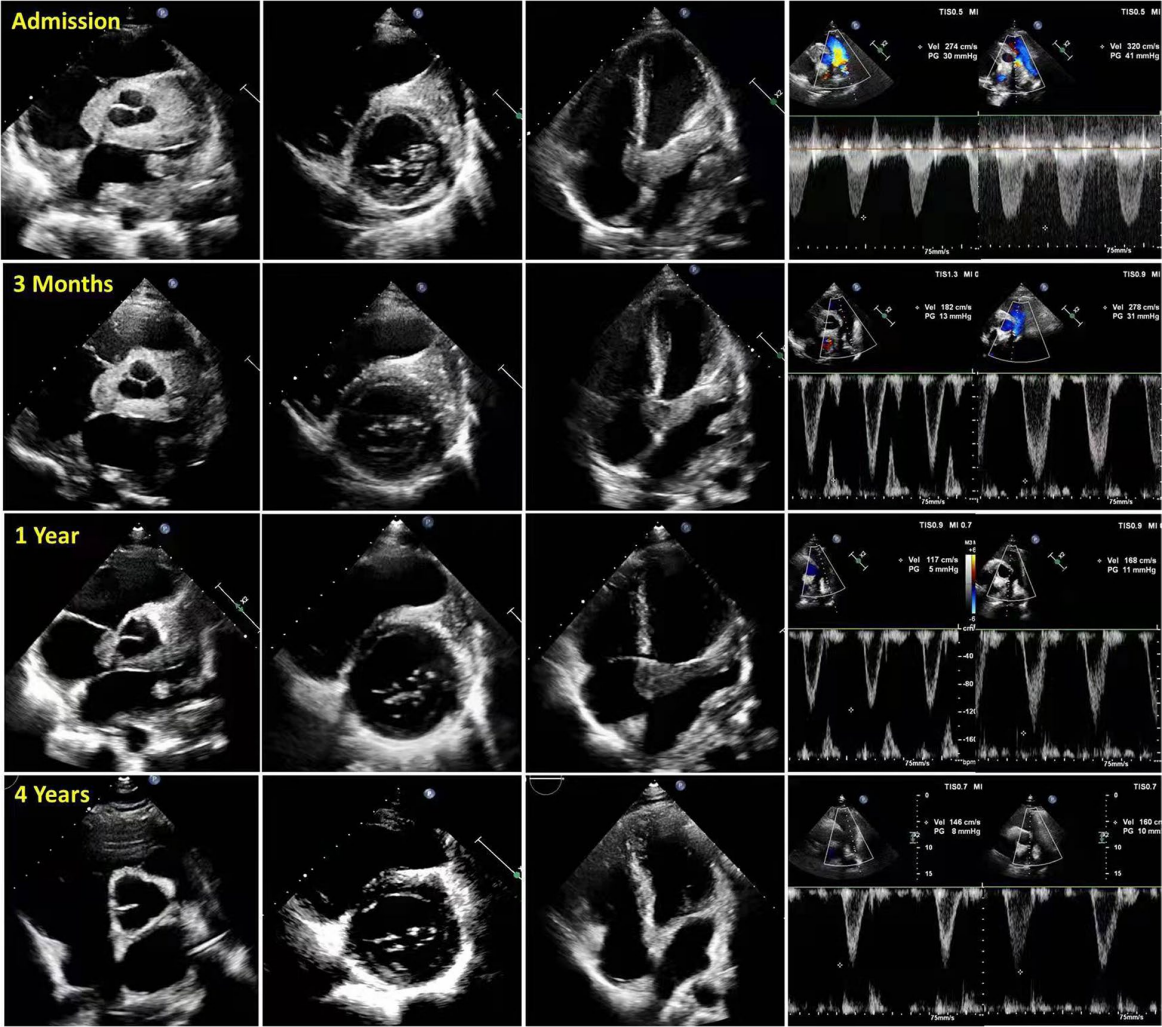
Echocardiography at admission and at different times after treatment showed the area of the lesion became significantly smaller and the blood flow velocity of the pulmonary artery branch returned to normal

Glucocorticoid is a standard treatment for FM. Thus, we initially prescribed 6 prednisone tablets (total dose: 30 mg) per day. We initially reduced the dose by 1 tablet every 3 months; when the dose was 15 mg per day, we reduced the dose by 1 tablet every 6 months. Eventually, we maintained the dose at 1 tablet (5 mg) per day. During the 4 years of follow-up, there was no recurrence of symptoms, the imaging findings revealed significant improvement (Fig. 2, Table 2, Video 1), and the laboratory results for CRP, ESR, and autoimmune indicators were all normal.

## Discussion

FM can be idiopathic or secondary, and most cases of secondary FM have infectious or non-infectious etiologies. The infectious etiologies include histoplasmosis, blastomycosis, coccidiomycosis, aspergillosis, and tuberculosis. The noninfectious etiologies include sarcoidosis, silicosis, rheumatoid arthritis, systemic lupus erythematosus, antineutrophil cytoplasmic antibody-associated vasculitis, IgG4-related disease, Riedel’s (fibrous) thyroiditis, and Behçet’s syndrome, tumor, and radiation or drug therapy [4–6]. We excluded secondary causes during the diagnosis, and the final diagnosis was idiopathic FM.

The two main imaging features of FM are local soft tissue masses around the mediastinum and diffuse mediastinal infiltration [7]. Diffuse mediastinitis can affect the large blood vessels of the heart. Li et al. reported a case of pulmonary vein stenosis caused by FM. Their color Doppler flow imaging (CDFI) showed a high-speed blood flow signal from the pulmonary vein in the left atrium, indicating pulmonary vein stenosis, but two-dimensional echocardiography did not directly show the lesions around the pulmonary vein [2]. Pereira-da-Silva et al. reported a case of FM with a mass in the right atrium and thickening of the aortic wall [3]. Their echocardiographic findings were similar to ours, but their echocardiography did not show a complete lesion.

The current case is very rare, in that the lesion had extensive invasion of the heart and had imaging features indicative of FM. Five specific features made our case unique. First, there was diffuse proliferation of fibrous tissue that invaded multiple chambers. Second, the lesion mainly invaded the large blood vessels of the heart, with further involvement of the atria and the ventricles. Third, the soft tissue mass was wrapped extensively along the anatomical structures of the heart, resulting in lumen stenosis. Fourth, the lesion protruded into the atrium and formed a soft tissue mass. Fifth, soft tissue masses infiltrated the myocardium. In addition, our CDFI showed a high-speed blood flow signal in the narrow pulmonary arteries and pulmonary veins.

The imaging findings of FM may suggest its etiology. Sherrick et al. reviewed the records of 33 patients with FM and found that the most likely etiology of the 27 localized cases was histoplasmosis, and the most likely etiology of the 6 diffuse cases was idiopathic or non-infectious [7]. Although there are some different interpretations, other studies reported diffuse cases without clear etiology. Our imaging features showed that the patient had diffuse disease, consistent with a diagnosis of idiopathic FM.

It is very rare for diffuse lesions to invade the heart, and a differential diagnosis to exclude tumors is necessary. Unfortunately, there are limited studies on the use of echocardiography to distinguish tumor from FM, especially because heart tumors are rare. We suggest two criteria to distinguish these two conditions. First, a malignant tumor mainly infiltrates the heart, but FM mostly encapsulates or wraps the heart. Second, a tumor may invade any part of the heart with no fixed pattern, but FM usually grows along the large blood vessels and then down to the atria and ventricles.

Encapsulated growth may also be encountered in Erdheim-Chester disease (ECD), whose imaging features are very similar to those of FM. ECD is a non-Langerhans histiocytosis of unknown origin. Its most common cardiac manifestation is a pseudotumor close to the right atrium that invades the right atrium and atrioventricular groove. Soft tissue masses also form around the aorta, pulmonary arteries, and their branching vessels [8]. Because FM and ECD are rare diseases with no specific imaging characteristics, histopathology is needed for a definitive diagnosis.

The diagnosis of FM is the most important element of the current case. Although CT and MRI can provide more comprehensive information, echocardiography has the advantages of being more readily available, less expensive, and radiation-free. Thus echocardiography may help in the diagnosis and management of FM. Although echocardiography does not eliminate the need for other imaging techniques, such as CT and MRI, it may allow clinicians to reduce their use during follow-up. The extensive cardiac involvement in the current case suggests this may be a prerequisite for the application of echocardiography. In addition, ultrasound has high resolution and provides a good visualization of soft tissue. Coupled with CDFI, it can be used to determine the possible nature of lesions. Although there are no echocardiographic criteria for the diagnosis of FM, our study shows that echocardiography can aid in the diagnosis of FM.

## Conclusions

Echocardiography can help in the characterization of the cardiac involvement of FM. It is helpful in the diagnosis, although histopathologic confirmation is mandatory, as well as for monitoring the progression of FM and the therapeutic responses to systemic therapies.

## Supplementary Information

**Additional file 1: Video 1.** Echocardiography showed the characteristics of FM invading the heart, as well as follow-up records.

## Data Availability

The datasets used and/or analysed during the current study are available. from the corresponding author on reasonable request.

## Abbreviations

CT: Computed tomography
MRI: Magnetic resonance imaging
CDFI: Color Doppler flow imaging
FM: Fibrosing mediastinitis
ECD: Erdheim-Chester disease
